# Supplementary material for: SOX4 exerts contrasting regulatory effects on labor-associated gene promoters in myometrial cells
Source: PLoS One. 2024 Apr 18;19(4):e0297847. doi: 10.1371/journal.pone.0297847 (PMC11025800; doi:10.1371/journal.pone.0297847)
Supplement: S1 File — (PDF) [file pone.0297847.s001.pdf]

Supporting Information for: SOX4 exerts contrasting regulatory effects on labor-associated gene promoters in myometrial cells.

Author List and Affiliations:

Nawrah Khader[1], Virlana M. Shchuka[1], Anna Dorogin[2,3], Oksana Shynlova[2, 3, 4], and Jennifer A. Mitchell[1*]

[1.] Department of Cell and Systems Biology, University of Toronto, Toronto, ON, Canada.

[2.] Lunenfeld Tanenbaum Research Institute, Sinai Health System, Toronto, ON, Canada.

[3.] Department of Obstetrics and Gynaecology, University of Toronto, Toronto, ON, Canada.

[4.] Department of Physiology, University of Toronto, Toronto, ON, Canada.

Current address: Department of Cell and Systems Biology, University of Toronto, Toronto, Canada

*Corresponding author: ja.mitchell@utoronto.ca (J.A.M)

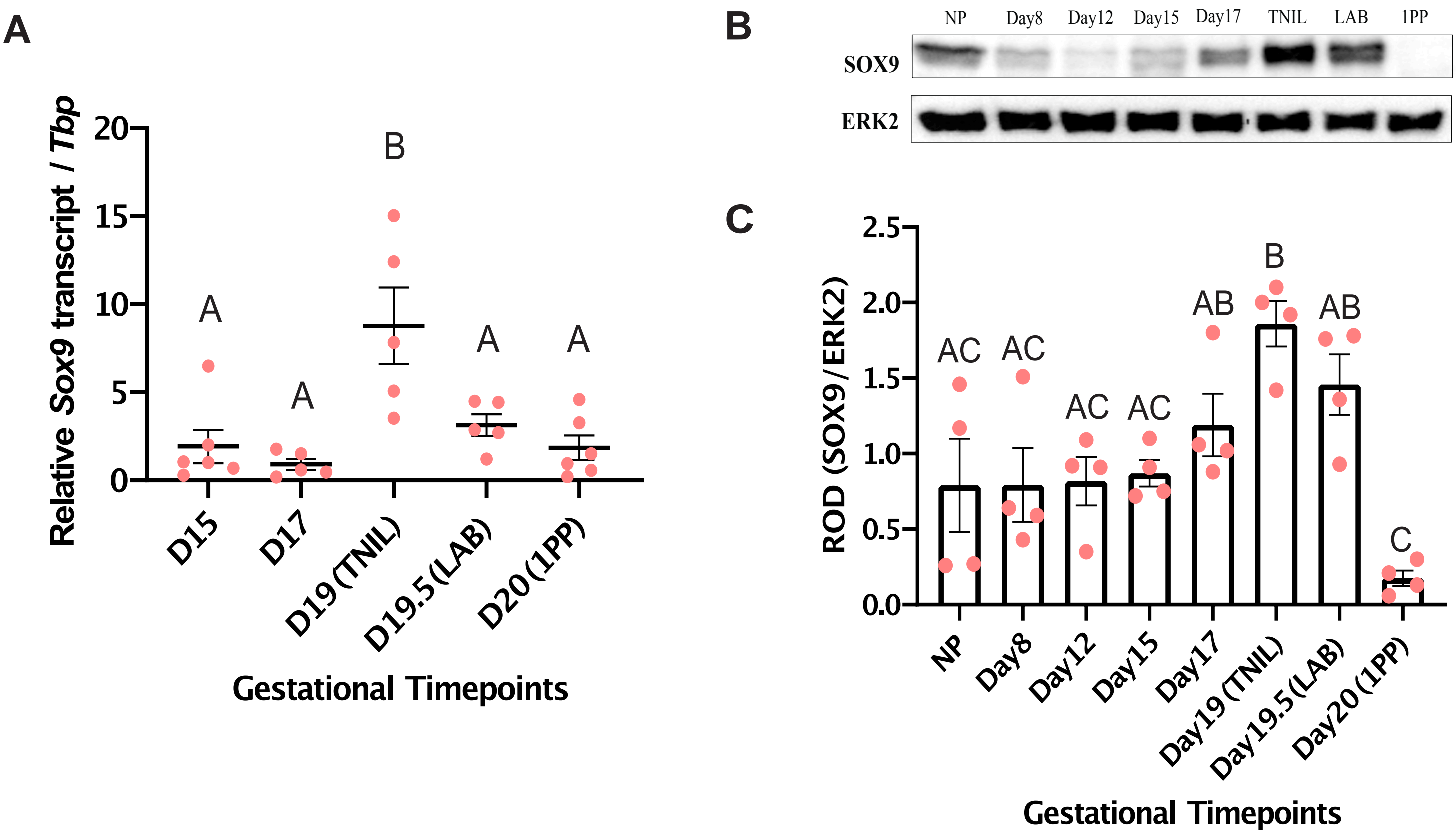

SFig 1. SOX9 gene and protein expression is significantly elevated in murine term myometrium tissues. (A) Transcript expression levels of *Sox9* was measured by RT-qPCR (±SEM) at indicated timepoints. Groups that exhibit significant differences ($p < 0.05$) as determined by one-way ANOVA are distinguished by different letters, while groups that do not show significant differences ($p > 0.05$) are labeled with the same letter.
(B) Representative immunoblot image of SOX9 and housekeeping protein ERK2 at gestational timepoints, indicated either by day (D) or status [term-not-in-labour (TNIL), labour (LAB), and postpartum (PP)].
(C) Densitometric analysis for SOX9 protein lysates extracted from mouse myometrium tissues at various gestational stages as indicated above. Groups that exhibit significant differences ($p < 0.05$) as determined by one-way ANOVA are distinguished by different letters, while groups that do not show significant differences ($p > 0.05$) are labeled with the same letter.

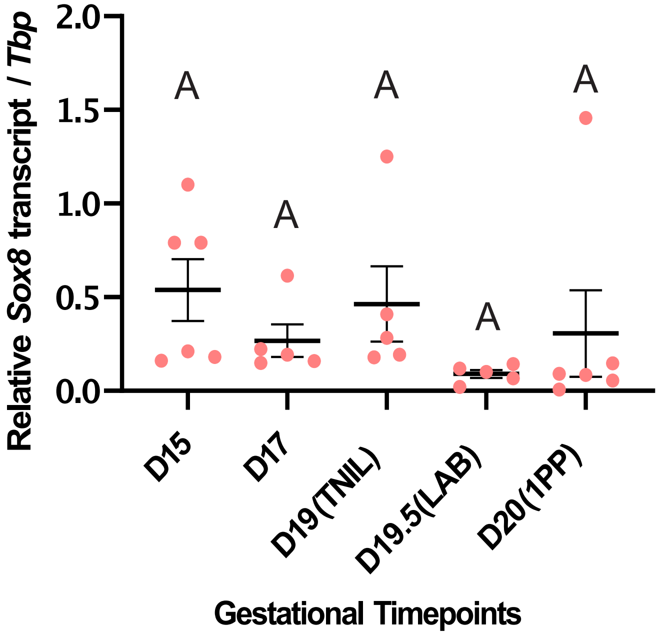

SFig 2. The *Sox8* gene expression is not differentially expressed in the murine myometrium tissues across gestation. Transcript levels of gene encoding the *Sox8* transcription factor, as measured by RT-qPCR (±SEM) at the designated timepoints.

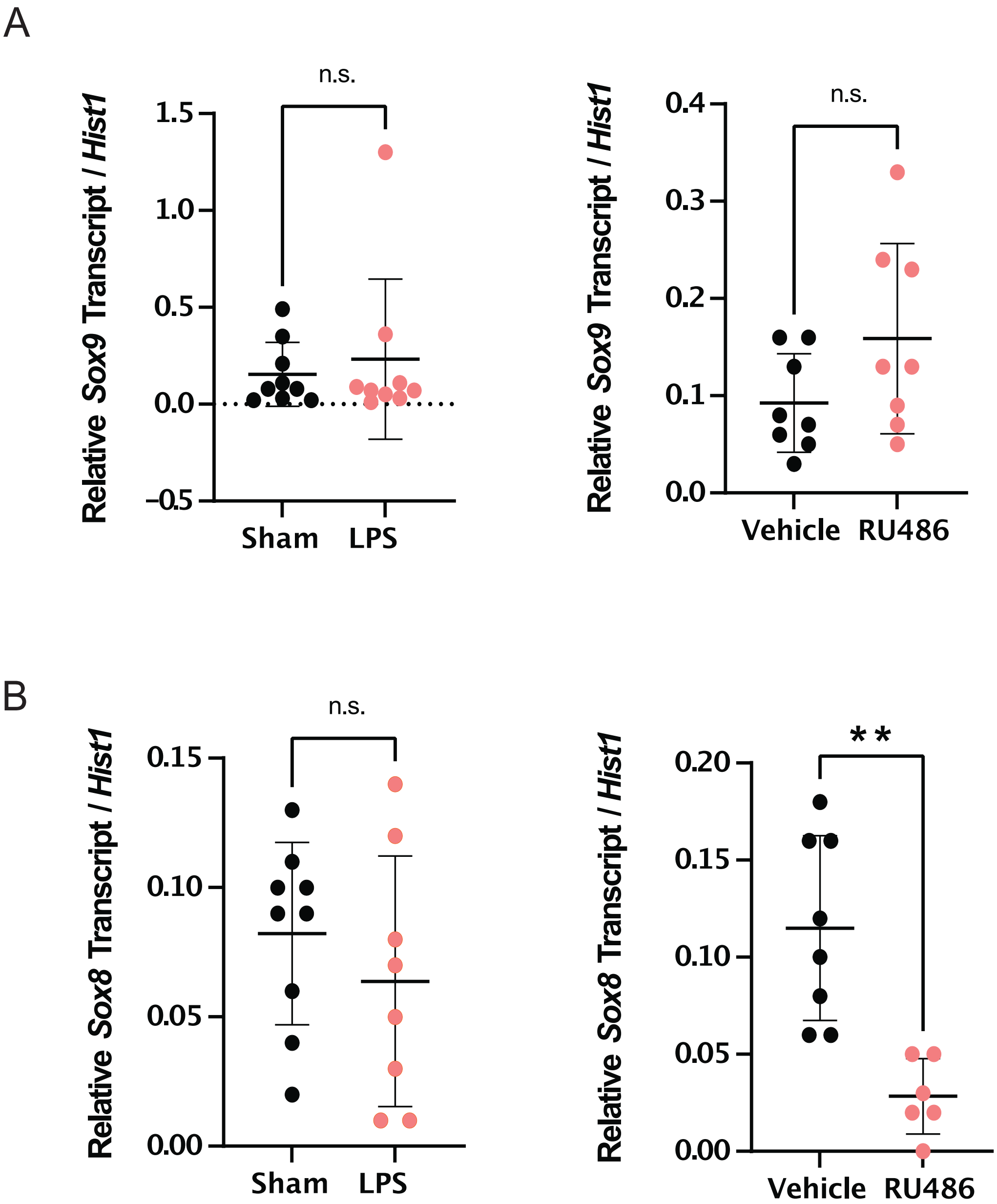

SFig 3. *Sox8* is up-regulated during the onset of the RU486-mediated but not during LPS-induced preterm labor. (A) Schematic of myometrium tissue collections at designated timepoints in two preterm labor induction models: local infection-simulation (LPS) and loss of progesterone (RU486).
Labour was induced by LPS (*left*) or RU486 (*right*), alongside sham and vehicle controls on day 15 and preterm labour occurred 18-24 hours post-injection as indicated by triangles. (B) Transcript levels of genes encoding the *Sox9* and *Sox8* transcription factors were measured by RT-qPCR (±SEM) at the timepoints corresponding to the schematic. Statistically significant differences in expression levels between control and preterm labor are marked by ** ($p < 0.01$).

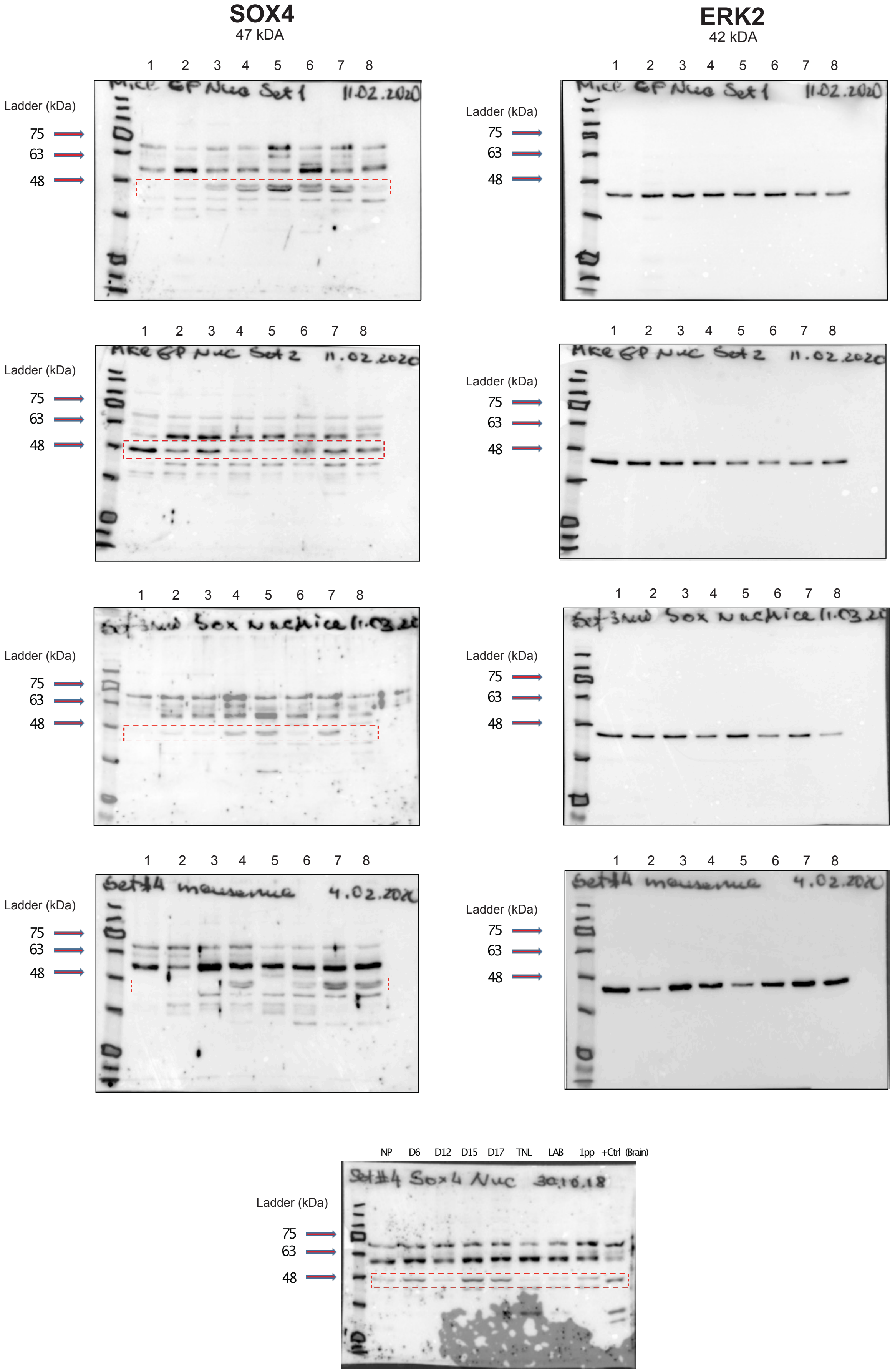

SFig 4. SOX4 (top) and ERK2 (bottom) original western blot images
Loading order is as follows: (1) Non-pregnant (2) Day 8 (3) Day 12 (4) Day 15 (5) Day 17 (6) term-not-in-labour (TNIL) (7) Labour (8) 1 day postpartum (D1PP). A representative initial trial western blot with myometrium tissues at different timepoints and a positive control (mouse brain tissue lysate) to ensure the identification of the correct band size (47 kDa) is also displayed [*bottom*].

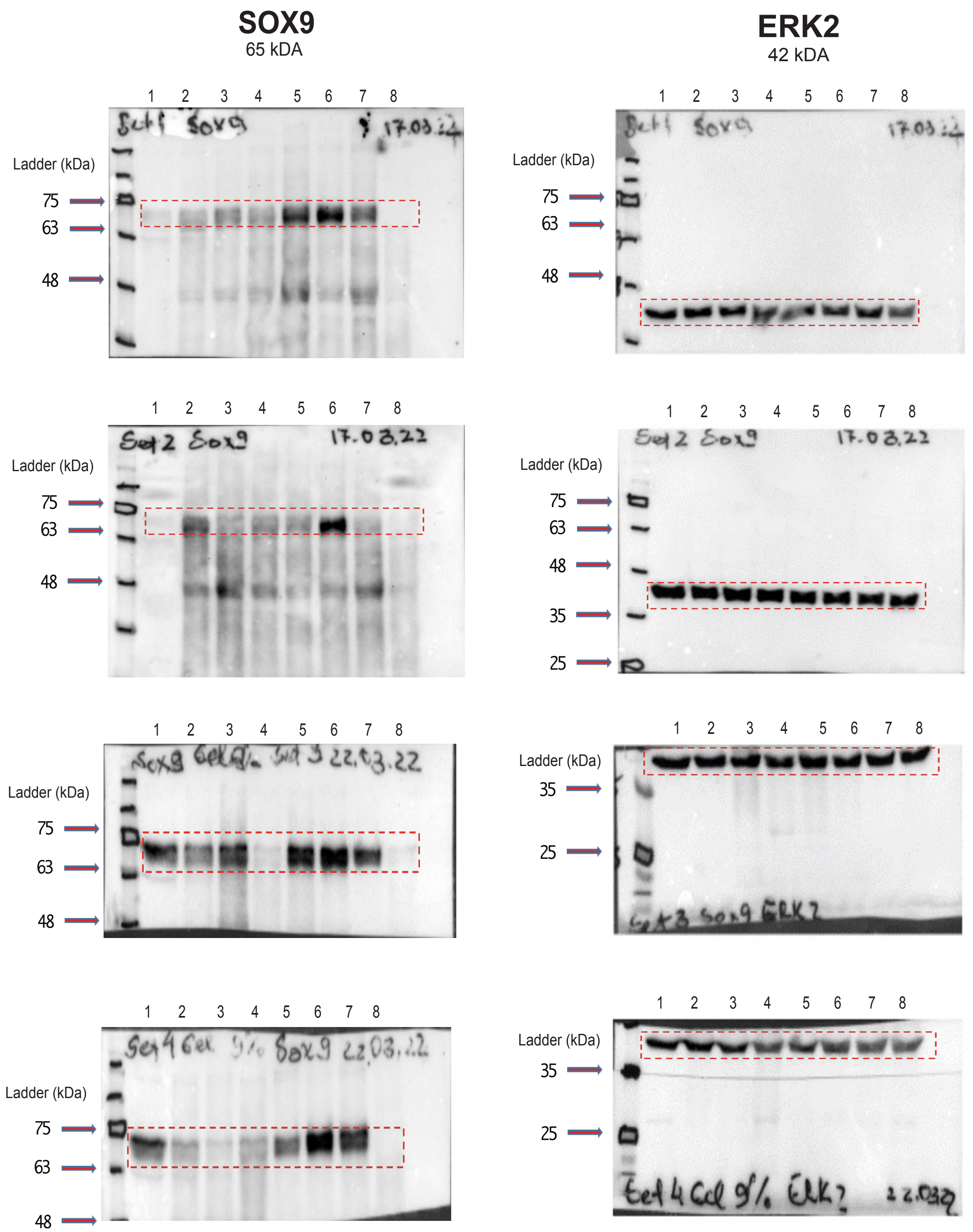

SFig 5. SOX9 (left) and ERK2 (right) original western blot images
Loading order is as follows: (1) Non-pregnant (2) Day 8 (3) Day 12 (4) Day 15 (5) Day 17 (6) term-not-in-labour (TNIL) (7) Labour (8) 1 day postpartum (D1PP).

SUPPLEMENTAL TABLES

S1 Table. List of primers used in mouse labor-upregulated TF expression quantification experiments.

TF Transcript Target	Forward Primer 5'->3'	Reverse Primer 5'->3'	Size (bp)
Sox4	TTGCCGACTTCACCTTCTTTC	GACAAGATTCCGTTCATCCAGC	104
Sox7	CCGACCTTCAGGGGACAAGA	ATCTTGCTGAGCTCCGCGTT	135
Sox8	CCGGCCAGTCTTCACACTCT	GCGAGAAGAGGCCCGTTTGTG	113
Sox9	AGCAGCGACGTCATCTCCAA	GCTGCTTCGACATCCACACG	184
Tbp	CTCAGTTACAGGTGGCAGCA	ACCAACAATCACCAACAGCA	186
Hist1	GGCCAAGGCTTCCAAGAAGT	CCACCTTGTAGTGGCTCTTGATA	137

S2 Table. List of primers used to clone labor-associated gene promoters into target reporter vectors for luciferase assays.

Promoter	Forward Primer 5'->3'	Reverse Primer 5'->3'	Size (bp)
Gja1	TCTCCTGAAGGAATGACCCATCCA	gtctgggcacctcTCTTTCACTTAATGAA AGTGAAGCC	503
Gja1 (cloning)	gaggatatcaagatctTCTCCTGAAGGA ATGACCCATCCA	ttggcatcttccatggGTCTGGGCACCTC TCTTTCACT	n/a
Fos	gaggatatcaagatctACTTATTTACAA TCCTTCACTTGCT	ttggcatcttccatggGGTCGAAGTTTGG GGAAAGCC	901
Ptgs2	AGCATTCCGATGAAGTGGAGCT	GGAGGTGGCAGTAGTGGTGG	757
Ptgs2 (cloning)	gaggatatcaagatctAGCATTCCGATG AAGTGGAGCT	ttggcatcttccatggGGAGGTGGCAGT AGTGGTGG	789
Mmp11	TGCCAAGTGTCAGTAGAGGTCAG	CTGCTGGGCCTGCTGGG	608
Mmp11 (cloning)	gaggatatcaagatctTGTGCCAAGTGT CAGTAGAGGT	ttggcatcttccatggTGCTGGGCCTGCT GGG	n/a
