# Supplementary material for: SOX4 exerts contrasting regulatory effects on labor-associated gene promoters in myometrial cells
Source: PLoS One. 2024 Apr 18;19(4):e0297847. doi: 10.1371/journal.pone.0297847 (PMC11025800; doi:10.1371/journal.pone.0297847)
Supplement: Attachment — Submitted filename: SOX4 Manuscript Rebuttal Letter Final.docx [file pone.0297847.s002.docx]

| **Date:** | Nov 22 2023 11:23AM |
| --- | --- |
| **To:** | "Jennifer A. Mitchell" ja.mitchell@utoronto.ca |
| **From:** | "PLOS ONE" plosone@plos.org |
| **Subject:** | PLOS ONE Decision: Revision required [PONE-D-23-29498] |

PONE-D-23-29498

SOX4 exerts contrasting regulatory effects on labor-associated gene promoters in myometrial cells.

PLOS ONE

Dear Dr. Mitchell,

Thank you for submitting your manuscript to PLOS ONE. After careful consideration, we feel that it has merit but does not fully meet PLOS ONE’s publication criteria as it currently stands. Therefore, we invite you to submit a revised version of the manuscript that addresses the points raised during the review process.

Please submit your revised manuscript by Jan 06 2024 11:59PM. If you will need more time than this to complete your revisions, please reply to this message or contact the journal office at [plosone@plos.org](mailto:plosone@plos.org). When you're ready to submit your revision, log on to https://www.editorialmanager.com/pone/ and select the 'Submissions Needing Revision' folder to locate your manuscript file.

Please include the following items when submitting your revised manuscript:

- A rebuttal letter that responds to each point raised by the academic editor and reviewer(s). You should upload this letter as a separate file labeled 'Response to Reviewers'.
- A marked-up copy of your manuscript that highlights changes made to the original version. You should upload this as a separate file labeled 'Revised Manuscript with Track Changes'.
- An unmarked version of your revised paper without tracked changes. You should upload this as a separate file labeled 'Manuscript'.

If you would like to make changes to your financial disclosure, please include your updated statement in your cover letter. Guidelines for resubmitting your figure files are available below the reviewer comments at the end of this letter.

If applicable, we recommend that you deposit your laboratory protocols in protocols.io to enhance the reproducibility of your results. Protocols.io assigns your protocol its own identifier (DOI) so that it can be cited independently in the future. For instructions see: <https://journals.plos.org/plosone/s/submission-guidelines#loc-laboratory-protocols>. Additionally, PLOS ONE offers an option for publishing peer-reviewed Lab Protocol articles, which describe protocols hosted on protocols.io. Read more information on sharing protocols at <https://plos.org/protocols?utm_medium=editorial-email&utm_source=authorletters&utm_campaign=protocols>.

We look forward to receiving your revised manuscript.

Kind regards,

Atsushi Asakura, Ph.D
Academic Editor
PLOS ONE

Journal Requirements:

1. When submitting your revision, we need you to address these additional requirements.

Please ensure that your manuscript meets PLOS ONE's style requirements, including those for file naming. The PLOS ONE style templates can be found at 
https://journals.plos.org/plosone/s/file?id=wjVg/PLOSOne_formatting_sample_main_body.pdf and 
https://journals.plos.org/plosone/s/file?id=ba62/PLOSOne_formatting_sample_title_authors_affiliations.pdf

2. Did you know that depositing data in a repository is associated with up to a 25% citation advantage (https://doi.org/10.1371/journal.pone.0230416)? If you’ve not already done so, consider depositing your raw data in a repository to ensure your work is read, appreciated and cited by the largest possible audience. You’ll also earn an Accessible Data icon on your published paper if you deposit your data in any participating repository (https://plos.org/open-science/open-data/#accessible-data).

3. Thank you for stating in your Funding Statement: 
This work was supported by the Canadian Institutes of Health Research (FRN 173252, held by J.A.M. and O.S.; cihr-irsc.gc.ca). Studentship funding was provided by Ontario Graduate Scholarship (OGS held by N.K.). The funders had no role in study design, data collection and analysis, decision to publish, or preparation of the manuscript.

Please provide an amended statement that declares all the funding or sources of support (whether external or internal to your organization) received during this study, as detailed online in our guide for authors at http://journals.plos.org/plosone/s/submit-now.  Please also include the statement “There was no additional external funding received for this study.” in your updated Funding Statement. 
Please include your amended Funding Statement within your cover letter. We will change the online submission form on your behalf.

4. PLOS ONE now requires that authors provide the original uncropped and unadjusted images underlying all blot or gel results reported in a submission’s figures or Supporting Information files. This policy and the journal’s other requirements for blot/gel reporting and figure preparation are described in detail at https://journals.plos.org/plosone/s/figures#loc-blot-and-gel-reporting-requirements and https://journals.plos.org/plosone/s/figures#loc-preparing-figures-from-image-files. When you submit your revised manuscript, please ensure that your figures adhere fully to these guidelines and provide the original underlying images for all blot or gel data reported in your submission. See the following link for instructions on providing the original image data: https://journals.plos.org/plosone/s/figures#loc-original-images-for-blots-and-gels. 
  
In your cover letter, please note whether your blot/gel image data are in Supporting Information or posted at a public data repository, provide the repository URL if relevant, and provide specific details as to which raw blot/gel images, if any, are not available. Email us at plosone@plos.org if you have any questions.

5. We note that Figures 4A and 4B in your submission contain copyrighted images. All PLOS content is published under the Creative Commons Attribution License (CC BY 4.0), which means that the manuscript, images, and Supporting Information files will be freely available online, and any third party is permitted to access, download, copy, distribute, and use these materials in any way, even commercially, with proper attribution. For more information, see our copyright guidelines: http://journals.plos.org/plosone/s/licenses-and-copyright.

We require you to either present written permission from the copyright holder to publish these figures specifically under the CC BY 4.0 license, or (2) remove the figures from your submission:
a. You may seek permission from the original copyright holder of Figures 4A and 4B to publish the content specifically under the CC BY 4.0 license. 
We recommend that you contact the original copyright holder with the Content Permission Form (http://journals.plos.org/plosone/s/file?id=7c09/content-permission-form.pdf) and the following text:
“I request permission for the open-access journal PLOS ONE to publish XXX under the Creative Commons Attribution License (CCAL) CC BY 4.0 (http://creativecommons.org/licenses/by/4.0/). Please be aware that this license allows unrestricted use and distribution, even commercially, by third parties. Please reply and provide explicit written permission to publish XXX under a CC BY license and complete the attached form.”
Please upload the completed Content Permission Form or other proof of granted permissions as an "Other" file with your submission.  
In the figure caption of the copyrighted figure, please include the following text: “Reprinted from [ref] under a CC BY license, with permission from [name of publisher], original copyright [original copyright year].”
b. If you are unable to obtain permission from the original copyright holder to publish these figures under the CC BY 4.0 license or if the copyright holder’s requirements are incompatible with the CC BY 4.0 license, please either i) remove the figure or ii) supply a replacement figure that complies with the CC BY 4.0 license. Please check copyright information on all replacement figures and update the figure caption with source information. If applicable, please specify in the figure caption text when a figure is similar but not identical to the original image and is therefore for illustrative purposes only.

6. Please include captions for your Supporting Information files at the end of your manuscript, and update any in-text citations to match accordingly. Please see our Supporting Information guidelines for more information: http://journals.plos.org/plosone/s/supporting-information.

*We have now added the caption at the end of the manuscript (line 596-598).*

[Note: HTML markup is below. Please do not edit.]

Reviewers' comments:

Reviewer's Responses to Questions

**Comments to the Author**

1. Is the manuscript technically sound, and do the data support the conclusions?

The manuscript must describe a technically sound piece of scientific research with data that supports the conclusions. Experiments must have been conducted rigorously, with appropriate controls, replication, and sample sizes. The conclusions must be drawn appropriately based on the data presented.

Reviewer #1: Partly

Reviewer #2: Partly

2. Has the statistical analysis been performed appropriately and rigorously?

Reviewer #1: I Don't Know

Reviewer #2: Yes

3. Have the authors made all data underlying the findings in their manuscript fully available?

The [PLOS Data policy](http://www.plosone.org/static/policies.action#sharing) requires authors to make all data underlying the findings described in their manuscript fully available without restriction, with rare exception (please refer to the Data Availability Statement in the manuscript PDF file). The data should be provided as part of the manuscript or its supporting information, or deposited to a public repository. For example, in addition to summary statistics, the data points behind means, medians and variance measures should be available. If there are restrictions on publicly sharing data—e.g. participant privacy or use of data from a third party—those must be specified.

Reviewer #1: Yes

Reviewer #2: Yes

4. Is the manuscript presented in an intelligible fashion and written in standard English?

PLOS ONE does not copyedit accepted manuscripts, so the language in submitted articles must be clear, correct, and unambiguous. Any typographical or grammatical errors should be corrected at revision, so please note any specific errors here.

Reviewer #1: Yes

Reviewer #2: Yes

5. Review Comments to the Author

Please use the space provided to explain your answers to the questions above. You may also include additional comments for the author, including concerns about dual publication, research ethics, or publication ethics. (Please upload your review as an attachment if it exceeds 20,000 characters)

Reviewer #1: SOX4 exerts contrasting regulatory effects on labor-associated gene promoters in myometrial cells. by Nawrah Khade et al.

This study has identified some SOX family members with enhanced expression in murine actively contractile myocytes in spontaneous and induced conditions. It could reveal novel roles for SOX4 in expressional regulation of “contractile” genes by luciferase assay. Mmp11 is found up-regulated by SOX4, while Ptgs2 is down-regulated. Though precise mechanisms remain to be elucidated, the study clarify some casts, including SOX4, involve drastic changes in laboring uterine myocytes.
The study obeys all the guidelines
The reviewer generally accepts the data presented and the discussion and conclusion from the data. The reviewer has some concerns below.

*We would like to thank the reviewer for their assessment of our work.*

Major points
1. Is Fig. 3B the typical blot of SOX4? It does not seem to match with the metric data of C. The quality of this Western analysis is low and better quality blot should be provided.

*RESPONSE: Figure 3 has now been updated with a higher quality blot of SOX4.*

2. The statistical analysis In Fig.4, there seems to be statistically significant differences. For example, B pG-FosP vs pG-FosP, SOX4, and C pG-Mmp11 vs pG-Mmp11, JUND/FOSL2. Please confirm again these points. The discussion and conclusion (Lines 241-244) may be needed to be modified when there be significant difference.

*RESPONSE: As seen in the statistical significance below (highlighted), Tukey's multiple comparisons test confirmed that these groups are* ***not statistically significant*** *which is reflected in our figure.*



| Tukey's multiple comparisons test | Mean Diff. | 95.00% CI of diff. | Below threshold? | Summary | Adjusted P Value |  |
| --- | --- | --- | --- | --- | --- | --- |
| pG-FosP vs. pG-FosP, JUND/JUND | -0.8934 | -2.768 to 0.9811 | No | ns | 0.6129 | A-B |
| pG-FosP vs. pG-FosP, JUND/FOSL2 | -9.601 | -11.48 to -7.726 | Yes | **** | <0.0001 | A-C |
| pG-FosP vs. pG-FosP, SOX4 | -1.470 | -3.344 to 0.4047 | No | ns | 0.1620 | A-D |
| pG-FosP vs. pG-FosP, JUND/JUND/SOX4 | -5.463 | -7.338 to -3.589 | Yes | **** | <0.0001 | A-E |
| pG-FosP vs. pG-FosP, JUND/FOSL2/SOX4 | -10.68 | -12.55 to -8.806 | Yes | **** | <0.0001 | A-F |
| pG-FosP, JUND/JUND vs. pG-FosP, JUND/FOSL2 | -8.707 | -10.58 to -6.833 | Yes | **** | <0.0001 | B-C |
| pG-FosP, JUND/JUND vs. pG-FosP, SOX4 | -0.5764 | -2.451 to 1.298 | No | ns | 0.8979 | B-D |
| pG-FosP, JUND/JUND vs. pG-FosP, JUND/JUND/SOX4 | -4.570 | -6.444 to -2.695 | Yes | **** | <0.0001 | B-E |
| pG-FosP, JUND/JUND vs. pG-FosP, JUND/FOSL2/SOX4 | -9.787 | -11.66 to -7.912 | Yes | **** | <0.0001 | B-F |
| pG-FosP, JUND/FOSL2 vs. pG-FosP, SOX4 | 8.131 | 6.256 to 10.01 | Yes | **** | <0.0001 | C-D |
| pG-FosP, JUND/FOSL2 vs. pG-FosP, JUND/JUND/SOX4 | 4.137 | 2.263 to 6.012 | Yes | **** | <0.0001 | C-E |
| pG-FosP, JUND/FOSL2 vs. pG-FosP, JUND/FOSL2/SOX4 | -1.080 | -2.954 to 0.7950 | No | ns | 0.4284 | C-F |
| pG-FosP, SOX4 vs. pG-FosP, JUND/JUND/SOX4 | -3.993 | -5.868 to -2.119 | Yes | *** | 0.0001 | D-E |
| pG-FosP, SOX4 vs. pG-FosP, JUND/FOSL2/SOX4 | -9.210 | -11.08 to -7.336 | Yes | **** | <0.0001 | D-F |
| pG-FosP, JUND/JUND/SOX4 vs. pG-FosP, JUND/FOSL2/SOX4 | -5.217 | -7.092 to -3.343 | Yes | **** | <0.0001 | E-F |

| Tukey's multiple comparisons test | Mean Diff. | 95.00% CI of diff. | Below threshold? | Summary | Adjusted P Value |  |
| --- | --- | --- | --- | --- | --- | --- |
| pG-Mmp11 vs. pG-Mmp11, JUND/JUND | -1.163 | -3.777 to 1.451 | No | ns | 0.6740 | A-B |
| pG-Mmp11 vs. pG-Mmp11, JUND/FOSL2 | -2.017 | -4.632 to 0.5966 | No | ns | 0.1726 | A-C |
| pG-Mmp11 vs. pG-Mmp11, SOX4 | -2.289 | -4.903 to 0.3254 | No | ns | 0.0997 | A-D |
| pG-Mmp11 vs. pG-Mmp11, JUND/JUND/SOX4 | -9.075 | -11.69 to -6.461 | Yes | **** | <0.0001 | A-E |
| pG-Mmp11 vs. pG-Mmp11, JUND/FOSL2/SOX4 | -9.367 | -11.98 to -6.753 | Yes | **** | <0.0001 | A-F |
| pG-Mmp11, JUND/JUND vs. pG-Mmp11, JUND/FOSL2 | -0.8545 | -3.469 to 1.760 | No | ns | 0.8731 | B-C |
| pG-Mmp11, JUND/JUND vs. pG-Mmp11, SOX4 | -1.126 | -3.740 to 1.488 | No | ns | 0.7009 | B-D |
| pG-Mmp11, JUND/JUND vs. pG-Mmp11, JUND/JUND/SOX4 | -7.912 | -10.53 to -5.298 | Yes | **** | <0.0001 | B-E |
| pG-Mmp11, JUND/JUND vs. pG-Mmp11, JUND/FOSL2/SOX4 | -8.204 | -10.82 to -5.590 | Yes | **** | <0.0001 | B-F |
| pG-Mmp11, JUND/FOSL2 vs. pG-Mmp11, SOX4 | -0.2713 | -2.885 to 2.343 | No | ns | 0.9991 | C-D |
| pG-Mmp11, JUND/FOSL2 vs. pG-Mmp11, JUND/JUND/SOX4 | -7.058 | -9.672 to -4.444 | Yes | **** | <0.0001 | C-E |
| pG-Mmp11, JUND/FOSL2 vs. pG-Mmp11, JUND/FOSL2/SOX4 | -7.350 | -9.964 to -4.735 | Yes | **** | <0.0001 | C-F |
| pG-Mmp11, SOX4 vs. pG-Mmp11, JUND/JUND/SOX4 | -6.786 | -9.400 to -4.172 | Yes | **** | <0.0001 | D-E |
| pG-Mmp11, SOX4 vs. pG-Mmp11, JUND/FOSL2/SOX4 | -7.078 | -9.692 to -4.464 | Yes | **** | <0.0001 | D-F |
| pG-Mmp11, JUND/JUND/SOX4 vs. pG-Mmp11, JUND/FOSL2/SOX4 | -0.2919 | -2.906 to 2.322 | No | ns | 0.9988 | E-F |

Minor points are
1. Is ERK2 the adequate marker of constantly expressed proteins by the myocytes? This enzyme can be phosphorylated. And the metric data of ERK blots included only unphosphorylated or the sum of un- and phosphorylated ones? The reviewer requests authors’ notice in the rebuttal or in the text.

***RESPONSE:*** *Expression levels of ERK1 and ERK2 protein remain unchanged and are therefore not gestationally regulated or affected by the labouring or postpartum processes (PMID: 12372814) and was confirmed by Li and colleagues (PMID: 12388473). The relative protein expression was normalized with ERK2 as it was the most stable protein expressed as previously determined and has been used in multiple recent publications for densitometric analysis using myometrial tissues in the rat (PMID: 17715430, 19602722, 28945005), mouse (PMID: 28945005, 34418351) myometrium throughout gestation and labour, as well as in the human myometrium (PMID: 29042599, 34114342), and primary human myometrial smooth muscle cells (PMID: 35011690). We chose not to use Actin and GAPDH as they are gestationally regulated in the rodent myometrium. For these reasons, we chose to normalize with total ERK2 using the sc-154 antibody as is now reflected in the manuscript.*

2. Indication of significant level of difference In Fig.4B, D is not needed. pG-Gja1P, SOX4 is AB, and pG-Gja1P, JUND/JUND/SOX4 is B. That is OK.

***RESPONSE:*** *We have now made the appropriate changes to Fig 4.*

3. Collection of myometrial samples (Line 408-410) Did authors accurately excise out the endometrial and stromal tissues from uterine samples?

***RESPONSE:*** *We previously specified the removal of the decidua (terminology for pregnant endometrium) however we have now added that the uterine stromal tissues were also removed (line 407-409).*

*The text now reads: “Both uterine horns were bisected longitudinally and dissected away from both pups and placentas and placed in ice-cold PBS. Through mechanical scraping on ice, the decidua, as well as the uterine stromal tissues, were carefully removed from the myometrium tissue.”*

4. What is the source of Syrian hamster myometrium (SHM)? There is no description in the text.

***RESPONSE:*** *We have added the source of the SHM cells in our text (line 427).*

*The text now read: “Syrian hamster myometrium (SHM) cells, generously supplied by Professor Oksana Shynlova..”*

5. It should be indicated in the figure legends or somewhere that error bars mean SD.

***RESPONSE:*** *We have made the appropriate changes in the figure legends (line 188 and 225) to indicate where data displayed use SEM for error bars (animal experiments) or SD (transfection experiments).*

Reviewer #2: In the current study, the authors investigated the roles of SOX family TFs in myometrial cells and found that Sox4 is elevated in murine myometrium during term labor and in two mouse models of preterm labor. Using luciferase reporter assay, they also revealed that SOX4 differentially affects labor associated gene promoter activity, in cooperation with AP-1 dimers. This study provides new clues that SOX4 paly a diverse role in regulating gene expression in the laboring myometrium. However, there are several issues to be further addressed.

*We would like to thank the reviewer for their assessment of our work.*

1. To confirm the effects of SOX4, and more important, to dissect the diverse effects of SOX4 on the different labor associated genes, the mRNA and protein expression of the target genes need to be determined after SOX4 was overexpressed in the cell model.

***RESPONSE:*** *We agree that to determine the role of SOX4 in labor-associated gene expression it would be appropriate to overexpress SOX4 in myometrial cells, however, this would best be done in a mouse model to assess the effect on pregnancy. Although the SHM cell model is routinely used for this type of reporter assay, as they are easy to transfect, it would not be the most appropriate situation in which to investigate the role of SOX4 on endogenous gene expression since these are hamster cells and we have been looking at the effect of SOX4 on mouse promoter driven expression. We have been quite careful in the text to ensure that we refer to the effect of SOX4 on promoter driven transcription and not refer to SOX4 as affecting endogenous mRNA or protein levels except in the discussion where we have proposed that this could be occurring.*

2. In figure 2A and 2B, the labels in the statistical diagram are not consistent with those in the schematic diagrams on the first lane, which causes confusion.

***RESPONSE****: We have now made the appropriate modification to Figure 2.*

3. Fig. 3A: why did the author use ERK2 as internal control, which is not a classical housekeeping gene, and may be substantially affected by laboring process?

***RESPONSE:*** *As stated above in response to reviewer 1:*

*Expression levels of ERK1 and ERK2 protein remain unchanged and are therefore not gestationally regulated or affected by the labouring or postpartum processes (PMID: 12372814) and was confirmed by Li and colleagues (PMID: 12388473). The relative protein expression was normalized with ERK2 as it was the most stable protein expressed as previously determined and has been used in multiple recent publications for densitometric analysis using myometrial tissues in the rat (PMID: 17715430, 19602722, 28945005), mouse (PMID: 28945005, 34418351) myometrium throughout gestation and labour, as well as in the human myometrium (PMID: 29042599, 34114342), and primary human myometrial smooth muscle cells (PMID: 35011690). We chose not to use Actin and GAPDH as they are gestationally regulated in the rodent myometrium. For these reasons, we chose to normalize with total ERK2 using the sc-154 antibody as is now reflected in the manuscript.*

*At line 455-456 the text now reads: “Membranes were then stripped and re-probed using an antibody that detects total ERK2 (sc-154) as it has shown to have the most stable protein expression in the myometrium (18,39)"*

4. In the previous study, the authors determined the Sox family expression in CD-1 mouse uterus, why didn’t they use the same cell model in the following luciferase reporter experiments, instead of Syrian hamster myometrium (SHM) cell?

***RESPONSE:*** *To our knowledge, the only mouse myometrial cell lines are the “SMU1-10” cells (PMID: 9170108) and m-M116 cells (PMID: 8641172) which have not been used in the context of reporter assays. Since none of these cells been transfected in the past, we are not in favour of purchasing this line (cost $6,376.50 USD for the SMU1-10 cells) to determine if they are transfectable. Additionally, our preliminary experiments have shown that transfection efficiencies of primary myometrial cell lines, both human and mouse are very low (~3-5%), which makes the experiment in these lines unfeasible in these cell lines as well.  SHM cells have been used extensively as a model system for reporter assays within a myometrial context (PMID: 36595497, PMID: 27220952, PMID: 15618352) and have a high transfection efficiency (see image below of transfection with pmaxGFP). Additionally, to stay consistent with our previously published findings that used SHM cells for MYB and ELF3 in the same context (PMID: 36595497), we chose to use these cells for our assays.*

6. PLOS authors have the option to publish the peer review history of their article ([what does this mean?](https://journals.plos.org/plosone/s/editorial-and-peer-review-process#loc-peer-review-history)). If published, this will include your full peer review and any attached files.


If you choose “no”, your identity will remain anonymous but your review may still be made public.


**Do you want your identity to be public for this peer review?** For information about this choice, including consent withdrawal, please see our [Privacy Policy](https://www.plos.org/privacy-policy).

Reviewer #1: No

Reviewer #2: **Yes:** Lu Gao

[NOTE: If reviewer comments were submitted as an attachment file, they will be attached to this email and accessible via the submission site. Please log into your account, locate the manuscript record, and check for the action link "View Attachments". If this link does not appear, there are no attachment files.]

While revising your submission, please upload your figure files to the Preflight Analysis and Conversion Engine (PACE) digital diagnostic tool, <https://pacev2.apexcovantage.com/>. PACE helps ensure that figures meet PLOS requirements. To use PACE, you must first register as a user. Registration is free. Then, login and navigate to the UPLOAD tab, where you will find detailed instructions on how to use the tool. If you encounter any issues or have any questions when using PACE, please email PLOS at [figures@plos.org](mailto:figures@plos.org). Please note that Supporting Information files do not need this step.

*In compliance with data protection regulations, you may request that we remove your personal registration details at any time.* [*(Remove my information/details)*](https://www.editorialmanager.com/pone/login.asp?a=r)*. Please contact the publication office if you have any questions.*
